# Intranasal Administration of RBD Nanoparticles Confers Induction of Mucosal and Systemic Immunity against SARS-CoV-2

**DOI:** 10.3390/vaccines9070768

**Published:** 2021-07-09

**Authors:** Tuksin Jearanaiwitayakul, Mathurin Seesen, Runglawan Chawengkirttikul, Jitra Limthongkul, Suttikarn Apichirapokey, Sompong Sapsutthipas, Supaporn Phumiamorn, Panya Sunintaboon, Sukathida Ubol

**Affiliations:** 1 Department of Microbiology, Faculty of Science, Mahidol University, Bangkok 10400, Thailand; tuksin.jear@gmail.com (T.J.); mathurin.jj@gmail.com (M.S.); runglawan.cha@mahidol.ac.th (R.C.); jitra.kas@mahidol.ac.th (J.L.); Suttikarn.api@gmail.com (S.A.); 2 Institute of Biological Products, Department of Medical Sciences, Ministry of Public Health, Nonthaburi 11000, Thailand; sompong.s@dmsc.mail.go.th (S.S.); supaporn.p@dmsc.mail.go.th (S.P.); 3 Department of Chemistry, Faculty of Science, Mahidol University, Salaya, Nakornpatom 73170, Thailand; panya.sun@mahidol.ac.th

**Keywords:** intranasal vaccine, RBD, COVID-19, nano-delivery vaccine

## Abstract

Mucosal immunity plays a significant role in host defense against viruses in the respiratory tract. Because the upper respiratory airway is a primary site of SARS-CoV-2 entry, immunization at the mucosa via the intranasal route could potentially lead to induction of local sterilizing immunity that protects against SARS-CoV-2 infection. In this study, we evaluated the immunogenicity of a receptor-binding domain (RBD) of SARS-CoV-2 spike glycoprotein loaded into *N,N,N*-trimethyl chitosan nanoparticles (RBD-TMC NPs). We showed that intranasal delivery of RBD-TMC NPs into mice induced robust local mucosal immunity, as evidenced by the presence of IgG and IgA responses in BALs and the lungs of immunized mice. Furthermore, mice intranasally administered with this platform of immunogens developed robust systemic antibody responses including serum IgG, IgG1, IgG2a, IgA and neutralizing antibodies. In addition, these immunized mice had significantly higher levels of activated splenic CD4^+^ and CD8^+^ cells compared with those that were administered with soluble RBD immunogen. Collectively, these findings shed light on an alternative route of vaccination that mimics the natural route of SARS-CoV-2 infection. This route of administration stimulated not only local mucosal responses but also the systemic compartment of the immune system.

## 1. Introduction

Severe acute respiratory syndrome coronavirus 2 (SARS-CoV-2) is a recent emerging virus that has spread globally and poses a serious threat to society, leading to the instability of healthcare systems worldwide. As of 5 June 2021, at least 171 million confirmed cases and 3.7 million deaths have been announced by the World Health Organization (WHO) (https://covid19.who.int/) (accessed on 5 June 2021).

SARS-CoV-2 is an enveloped positive-sense single-stranded RNA virus belonging to the *Betacoronavirus* genus [1]. SARS-CoV-2 infection can result in a wide range of clinical outcomes from asymptomatic infection and a mild illness to a life-threatening manifestation. The severe disease is known as acute respiratory distress syndrome (ARDS), which is a disease associated with pneumonia, manifesting the signs of shortness of breath and hypoxia [2]. Individuals with immune impairment or underlying health problems, or aged over 60 years are at greater risk for progression to respiratory failure [3]. To date, there is a shortage of approved specific therapeutic drugs against SARS-CoV-2. As the virus continues to spread rapidly across the world, build-up of herd immunity through mass immunization represents the most promising approach to confining this pandemic.

SARS-CoV-2 mainly targets the epithelial cells lining mucosal surfaces. Entry of SARS-CoV-2 initially occurs through binding of the viral spike glycoprotein (S) to the host receptors (e.g., angiotensin-converting enzyme 2 (ACE2)) and cellular transmembrane protease serine 2 (TMPRSS2). This interaction primes the S-glycoprotein into its functional form [4,5]. Upon exposure to host furin, S-glycoprotein is cleaved into two subunits, S1 and S2 [6]. The receptor-binding domain (RBD), which serves as a viral attachment factor, is located on S1, while S2 harbors two important peptide repertoires, heptad repeat 1 (HR1) and heptad repeat 2 (HR2) [7,8]. These regions play a key role in recognizing host receptors and mediating the fusion of the viral envelope and host membrane, respectively. Epitope mapping of the S-glycoprotein revealed the RBD as a major target for neutralizing antibodies, and identified many peptides on this protein as dominant T cell epitopes [9,10,11,12], suggesting that RBD may be a promising candidate for a SARS-CoV-2 vaccine.

Current SARS-CoV-2 vaccines approved for human use are administered via intramuscular injections. The natural route of SARS-CoV-2 infection occurs through the respiratory tract; thus, the mucosal immune response is a first-line defense against SARS-CoV-2 [13]. Therefore, mucosal vaccination via the intranasal route may offer an advantage in inducing sterilizing and localized mucosal immunity. Despite recent efforts to apply this strategy, the efficacy of intranasal vaccines has been largely hindered by several factors, including the nature of the viral antigens and the physical barriers of the mucosa, which lead to inefficient antigen permeation and uptake. These inherent limitations can be overcome by incorporating an adjuvant that possesses an immune modulator and a delivery system. Transportation of target immunogens across the nasal epithelial barrier enhances antigen presentation to immune cells at the nasal-associated lymphoid tissues (NALTs) of the upper respiratory tract (URT), which serve as inductive sites for the mucosal immune system [14].

To this end, nanoparticle-based formulations have recently emerged as an innovative strategy for vaccine antigen delivery to mucosal sites [15]. Among the nanoparticles used in the development of intranasal vaccines, chitosan and its derivatives have gained the most attention for their applications against infectious pathogens of the respiratory system [16,17,18]. Compared with other mucosal adjuvants, chitosan-based nanoparticles exert their effects through various mechanisms. For instance, this NPs acts as a vehicle that protects the vaccine from degradation by tissue-specific protease and releases the vaccine antigens in a sustained manner into the nasal cavity. In addition, chitosan harboring a mucoadhesive property enhances the adherence of NPs to the mucus layer, resulting in an increased residence time of the vaccine [19]. Furthermore, the surface moieties of chitosan determine the recognition and uptake by antigen-presenting cells (APCs) [20,21]. We previously reported that incorporation of influenza HA2/NP antigens into *N,N,N*-trimethyl chitosan nanoparticles (HA2/NP TMC NPs) could increase the antigen uptake by primary human nasal epithelial cells [22]. Moreover, TMC NPs significantly improved encapsidated HA2/NP antigenicity by enhancing the secretion of innate immune mediators that promoted APC activation and exerted antiviral activity that suppressed viral multiplication in an in vitro challenge assay [22].

To assess whether this vaccine platform can be adapted for generating a novel intranasal SARS-CoV-2 vaccine candidate, the recombinant RBD protein, which was selected as an immunogen, was produced and packaged into a TMC nanoparticle (RBD-TMC NP)-based delivery system. The ability of RBD-TMC NPs to stimulate localized mucosal and systemic immune responses through intranasal vaccination was evaluated in mouse models.

## 2. Materials and Methods

### 2.1. UV-Inactivated SARS-CoV-2

SARS-CoV-2 (hCoV-19/Thailand/74/2020) was isolated from a clinical specimen of a confirmed COVID-19 patient at the National Institute of Health, Department of Medical Sciences, Thailand. The working virus seed was cultivated in Vero cells. The inactivated viruses were prepared via exposure to UV-A irradiation for 30 min and used in capture ELISA.

### 2.2. Animals

Female BALB/c mice (6–8 weeks old) were purchased from Nomura Siam International (Nomura Siam International Co., Ltd). Mice were acclimated in an animal facility for a week prior to initial use. The protocol for the animal experimentations was approved and performed in accordance with the Faculty of Science, Mahidol University–Institutional Animal Care and Use Committee (MUSC–IACUC, protocol number: MUSC63-012-520).

### 2.3. Production of the SARS-CoV-2 RBD Antigen

The nucleotide sequence of the RBD region on the SARS-CoV-2 spike glycoprotein (SARS-CoV-2 RBD, GenBank accession number: NC_045512.2) was codon-optimized for expression in *P. pastoris*. The gene fragment flanked by the *XbaI* and *KpI* restriction sites was cloned into the pPICαβ expression vector (Invitrogen) before being electroporated into *P. pastoris*. The purification of recombinant protein in the culture supernatant was subsequently carried out using affinity chromatography on Ni^2+^conjugated chelating resin (Invitrogen) under native conditions. Further, the purified RBD was analyzed by immunoblotting analysis using mouse anti-His (C-term) antibody (Invitrogen) and rabbit anti-SARS-CoV-2 RBD polyclonal antibody (Sino Biological, China). The contaminated endotoxin was detected by a limulus amebocyte lysate (LAL) assay (Sigma Aldrich).

### 2.4. Preparation and Characterization of RBD Loaded in N,N,N-trimethyl Chitosan Nanoparticles (RBD-TMC NPs)

The RBD-loaded nanoparticles were prepared by ionotropic gelation as previously described [23]. Briefly, a sodium tripolyphosphate (TPP, 0.167 mg/mL) solution mixed with RBD protein (0.3 mg/mL) was added dropwise into a TMC solution in a HEPES buffer (pH 7.4) containing 1% (*v/v*) Tween 80 under continuous stirring for 1 h at room temperature. Subsequently, the unbound protein was separated from the encapsidated proteins using centrifugation at 10,000 ×g for 10 min on a glycerol bed. The supernatant was harvested for estimating the amount of unbound RBD by Pierce™ BCA Protein Assay Kit (Thermo Fisher Scientific, Rockford, IL, USA). The efficiency of protein loading in TMC NPs was calculated as described previously [23]. To characterize the physical properties of NPs, the mean of particle size, polydispersity index (PDI) and the zeta potential were evaluated by a zetasizer (Malvern Instruments Ltd., Malvern, UK).

### 2.5. In Vivo Immunization and Specimen Collection

Mice were intranasally immunized with RBD-TMC NPs containing 10 or 20 μg RBD/dose. The soluble RBD (sRBD) at 20 μg/dose was chosen as an antigen control. The mice immunized with 1× PBS or empty TMC NPs were used as control groups. Mice were intranasally immunized on Days 0, 8, 15 and 30 with 20 μL of the vaccine per dosage. Blood samples were collected on Days 7, 14, 29 and 45. On Days 30 and 45, mice were euthanized and specimens of the immunized mice, including blood, bronchoalveolar lavage (BAL), lungs and spleens, were harvested.

### 2.6. Quantitation of Antibody Titers

The levels of SARS-CoV-2 RBD-specific IgG, IgG1, IgG2a and IgA isotype antibodies present in sera, lung homogenates and BALs were quantitated using indirect ELISAs. Briefly, 96-well microplates were pre-coated with purified RBD antigen (1 μg/mL) at 4 °C for 16 h. The plates were washed with a washing buffer (0.05% Tween-20 in PBS, PBST) and further incubated with 1% BSA (*w/v*) in PBST for 1 h at room temperature. Twofold serial dilutions of sera, BALs and lung homogenates (100 μL/well) were added to each well and incubated for 2 h at room temperature before being incubated with the goat anti-mouse IgG (Invitrogen, Carlsbad, CA, USA), IgG1, IgG2a or IgA antibody-conjugated HRP (Southern Biotech, USA) for 2 h to detect RBD-specific antibodies. After incubation, TMB substrate was added, and the reaction was terminated using a 1 N HCl solution. The absorbance was then read at 450 nm using an ELISA reader. The cut-off endpoint titers (EPT) of RBD-specific antibodies were reported as the reciprocal of the highest dilution of samples that generated an absorbance greater than the threefold OD value of the blank controls.

### 2.7. Whole Virion Capture ELISA

Microtiter plates were treated with rabbit SARS-CoV-2 RBD polyclonal antibody (1:4000, Sino Biological, Beijing, China) overnight at 4 °C. The plates were washed with PBST and then blocked with 1% BSA (*w/v*) in PBST for 1 h at room temperature. After incubation, UV-inactivated SARS-CoV-2 (10⁴ PFU/well) was added and incubated for 2 h, followed by the addition of sera from immunized mice (dilution at 1:50). HRP-conjugated goat anti-mouse IgG antibody (1:3000, Invitrogen) was used for detection of SARS-CoV-2-specifc antibodies.

### 2.8. In Vitro Virus Neutralization Assay

Levels of neutralizing antibody were determined by plaque reduction neutralization assay (PRNT). Briefly, mouse sera were serially diluted fourfold in MEM supplemented with 2% FBS prior to mixing with equal volumes of SARS-CoV-2 (10^2^ PFU). The virus–antibody mixture was further incubated at 37 °C for 1 h. After incubation, the mixtures were inoculated into a monolayer of Vero cells. Virus adsorption was carried out at 37 °C for 1 h with gentle rocking. The overlay medium containing 1.2% of methyl cellulose with 10% FBS was added, and the plates were cultured at 37 °C with 5% CO_2_ for 6-7 days. At the end of incubation, cells were fixed and stained with 0.5% crystal violet in PBS. The number of plaques was counted and the percentage of plaque reduction at 50% was calculated compared with the virus control (virus alone).

### 2.9. Detection of RBD-Specific IgA Secreting Cells

The number of RBD-specific IgA secreting cells was enumerated by ELISPOT assay as previously described [24]. Briefly, MultiScreen IP filter plates (96-well) (Millipore, Billerica, MA, USA) were coated with RBD protein (2 μg/well) in PBS at 4 °C overnight. After washing, the plates were blocked with 200 μL/well of RPMI 1640 medium supplemented with 10% FBS for 2 h at room temperature. Afterward, threefold dilutions of splenocytes isolated from immunized mice were added into each well and cultivated at 37 °C with 5% CO_2_ for 5 h. After incubation, the cells were removed and the plates were washed with PBST 3 times. To detect IgA-secreting cells, HRP-conjugated goat anti-mouse IgA (Southern Biotech, Birmingham, AL, USA) was added and incubated for 1 h at room temperature. After 3 washes, the signals were developed by staining with DAB (SigmaFast DAB tablet, Sigma, St. Louis, MO, USA). The spots were scanned and counted on an ImmunoSpot S6 Ultimate Reader.

### 2.10. Ex Vivo Stimulation of Splenic Lymphocyte

Splenocytes (10^7^ cells/well) were cultivated in the presence of RBD (10 μg/mL) as a specific antigen or Con A (20 μg/mL) as a positive control. Unstimulated samples were used as a negative control. The stimulated cells were grown at 37 °C for 72 h and then incubated for an additional 5 h with Brefeldin A (BioLegend, San Diego, CA, USA). After incubation, the stimulated cells were harvested, treated with TruStain FcX (Anti-mouse CD16/32 antibody, BioLegend, San Diego, CA, USA) and subjected to surface staining with PE-Cy7 anti-mouse CD3, PE anti-mouse CD4 and FITC anti-mouse CD8 (BD Biosciences) antibodies. Subsequently, cells were processed with a fixation/permeabilization kit (BD Biosciences, San Diego, CA, USA), followed by staining with APC anti-mouse IFN-γ (BioLegend, San Diego, CA, USA). The percentages of positive cells were enumerated by flow cytometry. Simultaneously, aliquots of the culture supernatant were harvested at 12, 24, 48 and 72 h of treatment, and were then subjected to IFN-γ, IL-2 and IL-4 detection using ELISA (BioLegend, San Diego, CA, USA)

### 2.11. Statistical Analysis

Results are presented as the mean ± standard error of mean (SEM). Statistical analysis was conducted using Student’s *t-*test for comparisons between 2 groups. A value of *p* < 0.05 was considered to be statistically significant.

## 3. Results

### 3.1. Characterization of RBD-TMC NPs

**Figure 1:**
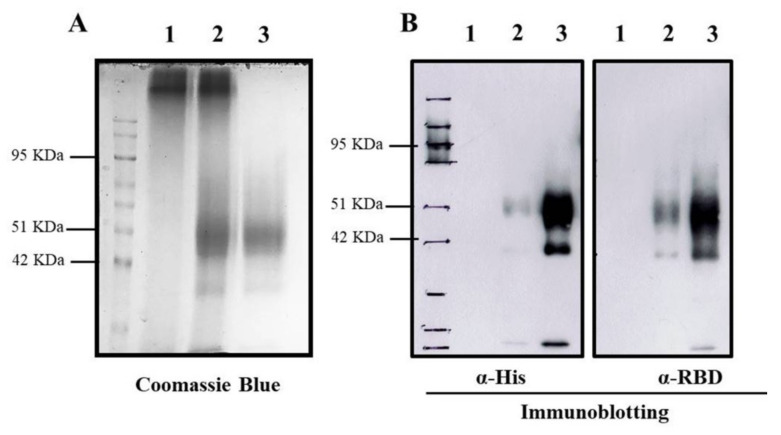
Entrapment of RBD protein in TMC NPs. The entrapped RBD protein in TMC NPs was detected by Coomassie Blue staining (**A**) and immunoblotting (**B**) using an anti-His tag (left) and anti-SARS-CoV2 RBD antibodies (right). 1: empty TMC NPs; 2: RBD-TMC NPs; 3: soluble RBD protein.

To encapsidate RBD protein into TMC NPs, the nanoparticles were generated using the ionotropic gelation method. This incorporation of protein into NPs was based on the electrostatic interaction of the positively charged TMC and the negatively charged sodium TPP, a cross-linker. This method yielded a nanoscale of NPs with a mean diameter of 386.5 ± 58.96 nm and a modest size distribution (0.407 ± 0.019), indicating a uniform mixture of particle sizes (Table 1). Additionally, the zeta potential of prepared NPs was +12.9 ± 0.651. This suggested that the NPs contained a cationic moiety on the surface (Table 1). We also found that RBD protein was efficiently entrapped into TMC NPs. This was demonstrated by successful loading efficiency as high as 99% (Table 1 and Figure 1A). The successful entrapment of RBD into NPs was also validated by immunoblotting. The results showed that encapsidated protein could be reacted with the tested antibodies (Figure 1B). Overall, these findings suggested that the ionotropic gelation method can be used to encapsulate the RBD antigen without interfering with RBD antigenicity.

### 3.2. RBD-TMC NPs Induce Mucosal Immunity in the Respiratory Tract

**Figure 2:**
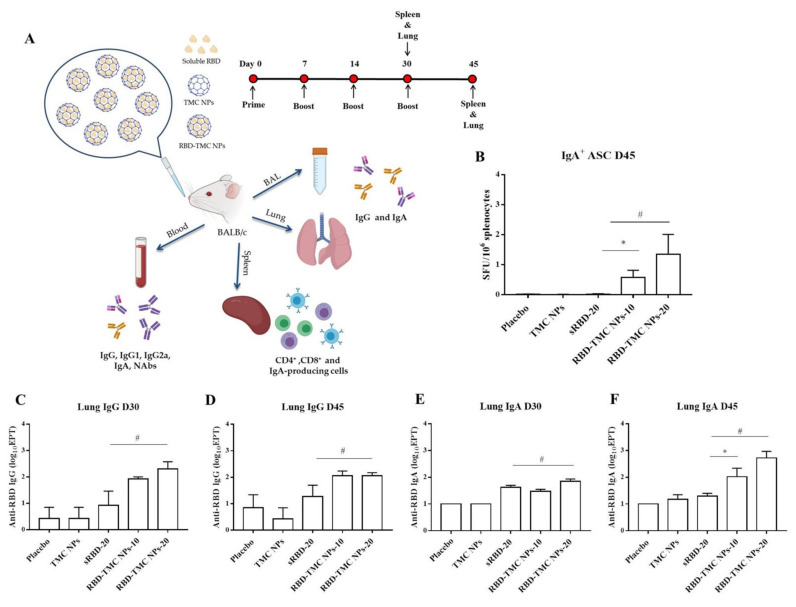
Mucosal immunization of nanoparticulated form of RBD robustly induced B cell-secreting IgA production and antibody responses in the lung. (**A**) Schematic representation of the study design. Mice were intranasally immunized with three or four doses of sRBD (20 μg/dose) or RBD-TMC NPs (10 or 20 μg/dose). After 14 days of three or four doses of immunization, the BALs, lungs and blood were harvested for assessment of the antibody responses. The cellular immune responses in the spleens of vaccinated mice were evaluated. (**B**) IgA-antibody secreting cells (ASC) specific to RBD in the spleens of mice on Day 45 were detected using ELISPOT. The levels of IgG and IgA in lung homogenates on Days 30 (**C**,**E**) and 45 (**D**,**F**) post-immunization were quantitated by indirect ELISA. Data are means ± SEM (*n* = 4–5). *, # indicates significant differences between soluble RBD and RBD-TMC NPs at 10 and 20 μg/dose, respectively (*p* < 0.05).

Mucosal secretory IgA mediates protection from SARS-CoV-2 infection in the nasal compartment [25,26,27]; as such, it is vital to understand the capacity of RBD-TMC NPs to elicit mucosal immunity. Mice were intranasally immunized and samples were harvested as shown in Figure 2A. We tracked the prevalence of RBD-specific IgA-secreting cells in the spleen of mice intranasally immunized with the vaccine using ELISPOT. As shown in Figure 2B, RBD-specific IgA secreting cells were massively generated in mice immunized with four doses of RBD-TMC NPs, but not with three doses (data not shown). In contrast, sRBD immunization failed to induce IgA-secreting cells (Figure 2B).

To investigate whether intranasal immunization with RBD-TMC NPs induced local mucosal immunity, IgA and IgG levels in the lung homogenates of immunized mice were quantified via ELISAs. As shown in Figure 2C,D, high titers of lung IgG were detected in mice after immunization with three and four doses of RBD-TMC NPs, while mice immunized with 20 μg sRBD developed significantly lower IgG titers. Similarly, increased IgA production could be detected after three vaccine doses (Figure 2E). After receiving four doses of immunization, the level of IgA production in mice immunized with encapsidated antigens (200 ± 149 and 940 ± 577 for 10 and 20 μg, respectively) was statistically higher than that of mice receiving the soluble form of antigens (21 ± 4) (Figure 2F).

**Figure 3:**
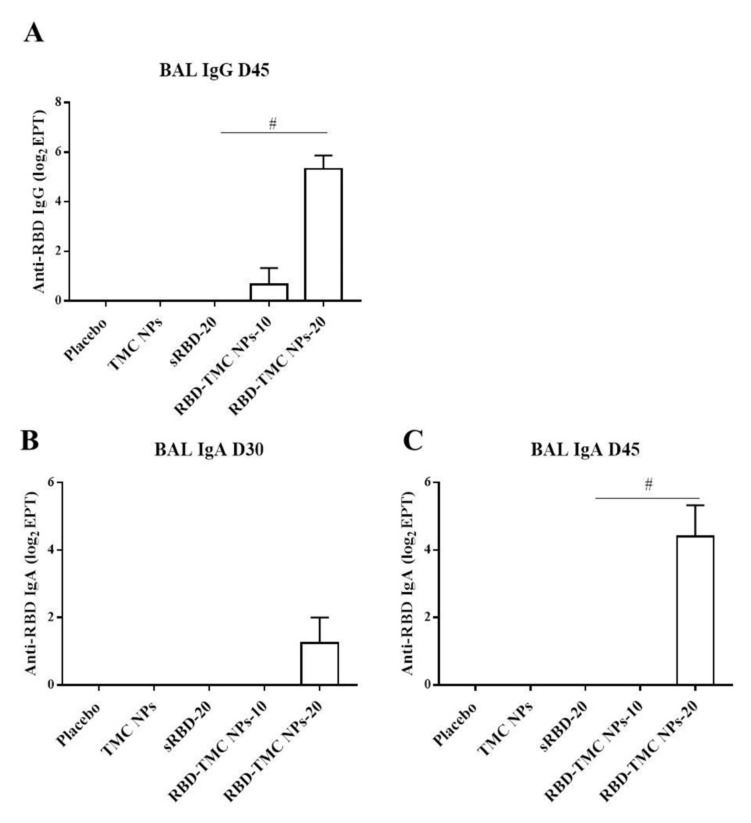
IgG and secretory IgA responses in BALs of immunized mice upon RBD-TMC NP vaccination. BALs were collected from immunized mice and subjected to quantitation of IgG on Day 45 (**A**) and secretory IgA on Days 30 (**B**) and 45 of immunization (**C**) using an indirect ELISA. The results show means ± SEM (*n* = 4–5). # indicates significant differences between soluble RBD and RBD-TMC NPs (*p* < 0.05).

The mucosal antibody response in BALs was also studied using ELISA. The results showed that IgG response was detectable from Day 30 or after three doses of immunization, in which one out of four mice immunized with either sRBD or RBD-TMC NPs produced BAL IgG at a dilution of 1:10 (data not shown). On Day 45, all mice receiving 20 μg of encapsidated RBD generated BAL IgG at a significantly higher level (56 ± 26) than mice in the control group and mice receiving other regimens of the immunogen (Figure 3A). A similar result was found for the production of secretory IgA, in which mice vaccinated with RBD-TMC NPs secreted a robust IgA response in BAL on Day 45 (44 ± 24) (Figure 3B,C). By contrast, sRBD could not activate the production of BAL IgA. These results suggested that TMC may act as a potent nasal adjuvant that enhances the local immune response toward RBD antigens.

### 3.3. RBD-Based TMC Nanoparticle Vaccine Augmented Systemic Humoral Responses

**Figure 4:**
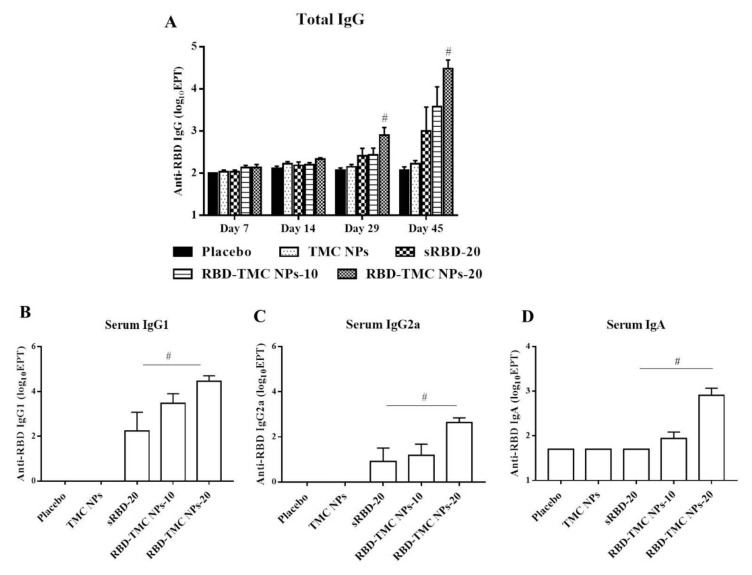
Intranasal administration with the nanoparticle form of RBD induced systemic antibody responses. Mice were vaccinated with sRBD or RBD-TMC NPs (10 or 20 μg/dose), and sera were harvested on Days 7, 14, 29 and 45 post-immunization for detection of RBD-specific IgG using indirect ELISA (**A**). On Day 45 after post-immunization, the levels of IgG1 (**B**), IgG2a (**C**) and IgA (**D**) of mouse sera were determined by indirect ELISA. Results are shown as means ± SEM (*n* = 4–8). # indicates significant differences between sRBD and RBD-TMC NPs (*p* < 0.05).

To study the potential effects of intranasal administration of RBD-TMC NPs on the production of systemic antibodies, levels of RBD-specific IgG in the sera of immunized mice were monitored by indirect ELISA. After three doses of vaccine immunization, the titers of IgG were significantly higher in mice that received 20 μg of RBD-TMC NPs (1600 ± 685) compared with mice immunized with sRBD (512.50 ± 237) at the same concentration. By Day 45, mice immunized with four doses of RBD-TMC NPs generated the highest titers of serum anti-RBD IgG, with a mean titer of 55,388 ± 32,726 and 58,000 ± 23,799 at 10 and 20 μg/dose, respectively (Figure 4A).

Typically, serum IgG1/IgG2a serves as an indirect marker of Th-1/Th-2 response, in which the production of IgG2a isotype is directed by Th-1 cytokines, while IgG1 production correlates with high levels of Th-2 cytokines [28,29]. We next profiled IgG isotypes in the sera of immunized mice by performing indirect ELISA to assess the immunomodulatory effects of RBD-TMC NPs on the Th-1/Th-2 axis. As depicted in Figure 4B, mice intranasally vaccinated with sRBD or RBD-TMC NPs elicited an RBD-specific IgG1 response. Likewise, immunization with RBD-TMC NPs or sRBD also resulted in the production of serum IgG2a (Figure 4C). Our findings imply that intranasal administration of RBD immunogens directed both Th-1 and Th-2 responses. 

As revealed in Figure 2B, RBD-TMC NPs strongly activated RBD-specific IgA-secreting cells. We therefore determined IgA production in blood circulation using ELISA. Figure 4D showed that the production of serum IgA was stimulated in mice following RBD-TMC NP vaccination. Conversely, sRBD was unable to induce serum IgA production, suggesting that TMC acts as an adjuvant boosting a systemic IgA response.

### 3.4. Neutralizing Activity of RBD-Specific Immune Sera against SARS-CoV-2

**Figure 5:**
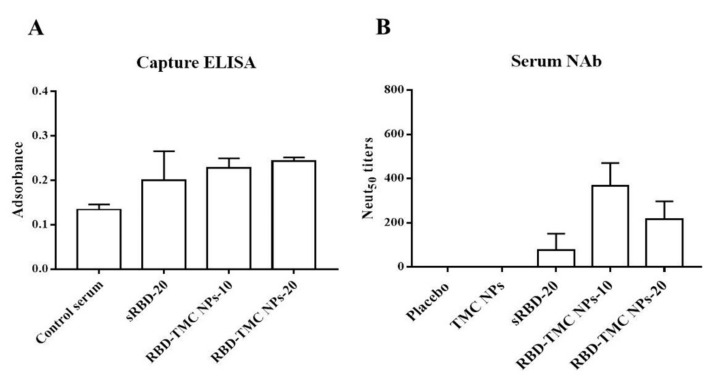
In vitro neutralization of sera against SARS-CoV-2. Sera from immunized mice on Day 45 were harvested. The binding of immunized sera (diluted 1:50) to UV-killed SARS-CoV-2 particles was determined by capture ELISA (**A**), and NAb titers were measured by PRNT (**B**). The results are means ± SEM (*n* = 4).

We further investigated whether the RBD-specific antibodies activated by RBD-TMC NP immunization were able to recognize and bind to SARS-CoV-2 particles. Capture ELISA was performed using UV-killed SARS-CoV-2 as an antigen. The results showed that the sera of mice immunized with RBD-TMC NPs strongly reacted with SARS-CoV-2 virions (Figure 5A). This suggested that RBD-specific antibodies induced by RBD-TMC NP immunization may neutralize SARS-CoV-2 infection.

This result led us to investigate whether RBD-TMC NP-induced antibody responses can protect against SARS-CoV-2 infection. To this end, we performed an in vitro neutralization assay using PRNT. By Day 45, all mice that received either 10 or 20 μg RBD-TMC NPs immunogens produced anti-SARS-CoV-2 neutralizing antibodies in their sera. The average PRNT titer was 366 ± 104 and 216 ± 81 for 10 and 20 μg of immunogens, respectively. For mice immunized with 20 μg of sRBD, only one out of four mice exhibited neutralizing activity (Figure 5B).

### 3.5. The Intranasal Nanoparticle Vaccine Enhanced the Systemic Cell-Mediated Immune Response

**Figure 6:**
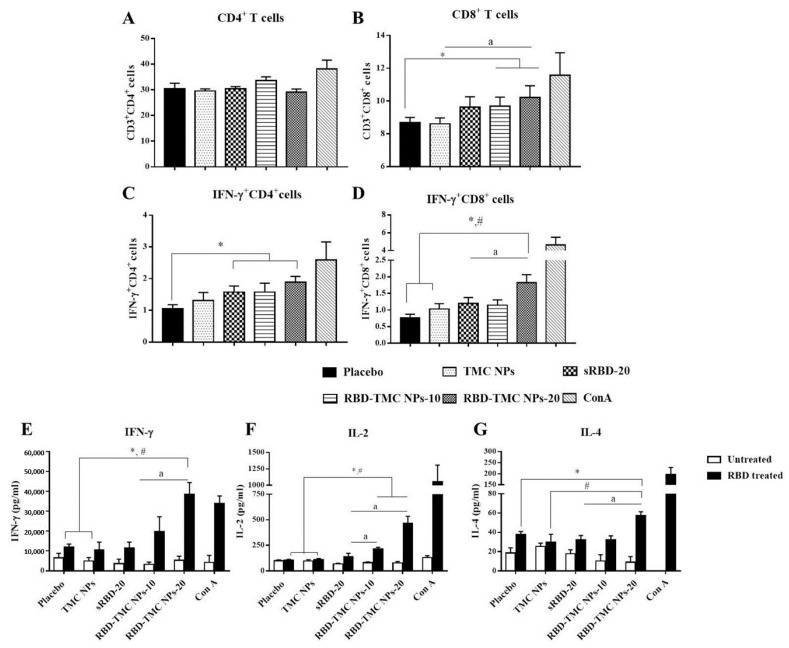
Robust cell-mediated immune responses after intranasal immunization with RBD-TMC NPs. Mice were vaccinated with sRBD or RBD-TMC NPs. By Day 45 of immunization, the splenocytes were isolated and stimulated with 10 μg/mL of RBD antigens for 72 h. ConA (20 μg/mL) was used as positive stimulation. The stimulated cells were subjected to quantitation of CD4^+^ (**A**), CD8^+^ (**B**), IFN-γ^+^CD4^+^ (**C**) and IFN-γ^+^CD8^+^ cells (**D**) using flow cytometry. The levels of cytokines including IFN-γ at 72 h (**E**), IL-2 at 24 h (**F**) and IL-4 at 48 h (**G**) in the culture supernatant of splenic lymphocytes were determined by ELISA. Data are presented as means ± SEM (*n* = 4–5). *, # indicates significant differences in the groups of mice receiving sRBD and RBD-TMC NPs compared with the placebo and TMC NPs, respectively. “a” indicates a significant difference between mice administered sRBD and RBD-TMC NPs (*p* < 0.05).

To evaluate whether administration of RBD-TMC NPs at the mucosal site could activate a cell-mediated immune response, spleens were harvested from immunized mice on Day 45 after immunization. Splenocytes were isolated and stimulated ex vivo, and the frequencies of CD4^+^, CD8^+^ and activated T cells (IFN-γ^+^CD4^+^ and IFN-γ^+^CD8^+^ cells) were monitored by flow cytometry. As illustrated in Figure 6A, RBD in its soluble and encapsidated forms both stimulated the expansion of CD4^+^ cells at the same level as the control groups, placebo and TMC NPs. Interestingly, sRBD and RBD-TMC NPs stimulated a significantly higher frequency of IFN-γ^+^CD4^+^ cells compared with the diluent control (Figure 6C). Notably, mice treated with RBD-TMC NPs significantly developed a greater frequency of CD8^+^ cells when compared with the control groups (Figure 6B). A similar pattern of results was detected for IFN-γ^+^CD8^+^ cell frequency. Among the groups of immunized mice, RBD-TMC NPs administered at 20 μg/dose stimulated the highest number of IFN-γ^+^CD8^+^ cells (Figure 6D). These results suggested that intranasal vaccination with RBD-TMC NPs activated a strong cell-mediated immune response.

The activation of T cell responses was further validated. It is widely accepted that IFN-γ and IL-2 cytokines are indicative markers of Th-1 polarization, whereas an induction of the Th-2 immune response is a unique immunological activity of IL-4. Levels of secreted IFN-γ, IL-2 and IL-4 in the supernatant of RBD-treated splenocyte cultures were determined at 12, 24, 48 and 72 h of treatment using ELISA. As expected, splenocytes isolated from RBD-TMC NP-vaccinated mice strongly responded to the RBD antigen through upregulation of IFN-γ, IL-2 and IL-4 production. Levels of IFN-γ, IL-2 and IL-4 production peaked at 72, 24 and 48 h of treatment, respectively (Figure 6E–G). By contrast, splenocytes from mice immunized with sRBD produced very low levels of these cytokines in response to RBD treatment (Figure 6E–G). This profile of cytokine responses suggested that RBD-TMC NPs strongly activate both Th-1 and Th-2 related cytokines. Taken together, the results of cellular cytokines and the percentage of CD4^+^/CD8^+^ responses indicated that RBD-TMC NPs strongly activate systemic cellular immunity.

## 4. Discussion

The mucosal immune response is a powerful arm of host immunity that prevents the entry of various types of pathogens at the mucosa. Currently, most SARS-CoV-2 vaccines used in human are administered via intramuscular injections. As such, myocytes are expected to be the major target of vaccine antigens. Unfortunately, myocytes are not professional antigen-presenting cells (APCs) and can present antigens exclusively through the MHC Class I pathway. In addition, myocytes are unable to migrate to draining lymph nodes, where immunological activation takes place. This means that the ability of myocytes to initiate adaptive immune responses is limited. Moreover, the respiratory tract is a common route of SARS-CoV-2 infection; thus, immunization at mucosal sites mimics this interaction and induces a highly specialized local immune response against SARS-CoV-2. Additionally, there are mucosal-resident dendritic cells, which serve as the principal immune cells that take up foreign antigens and mount local immune responses within mucosa-associated lymphoid tissues (MALTs) [30,31]. The role of mucosal immunization in defense against SARS-CoV-2 infection is supported by a previous report that intranasal vaccination with an adenovirus-based vaccine offered superior mucosal immunity compared with intramuscular injection, protecting from SARS-CoV-2 infection in both the upper and lower respiratory tracts [32]. Moreover, it is widely accepted that the anatomical and physiochemical barriers of the mucosa can determine vaccine efficacy [33]. Thus, vaccines that facilitate antigen adsorption and generate a robust immune response would greatly advance the field of intranasal vaccine development.

In this study, we developed a potential intranasal vaccine candidate based on the incorporation of RBD glycoprotein into TMC NPs (RBD-TMC NPs). Similar to our previous reports [22,23], the use of ionotropic gelation enabled a high RBD packing efficiency without any evidence of protein damage. The generated NPs had a size of less than 500 nm and a cationic charge, optimizing uptake by APCs [34]. Furthermore, NPs that possessed a positive charge could easily be deposited on a negatively charged mucosal layer via ionic bonding. This, in turn, promoted antigen uptake by cells of the mucosal layer and/or penetration of antigenic materials by transiently opening the tight junction of mucosal epithelial cells [22,35].

Our approach supports the use of TMC in improving antigen immunogenicity in a model of intranasal administration [17,36]. We showed here that intranasal vaccination with RBD-TMC NPs led to more robust production of mucosal IgG and IgA compared with sRBD immunization. The mucosal antibody response may be mediated by the immunostimulatory and delivery effects of TMC NPs. This type of nanoparticle has a mucoadhesive characteristic, meaning that it can prolong the residence time of antigens in the nasal cavity and, in turn, enhance uptake by nasal epithelial cells, local APCs and M-cells [22,37]. Among the Ig isotypes elicited by intranasal vaccination, secretory IgA (sIgA) had a significant role in preventing viral infection at the mucosal surface. This is supported by the correlation between sIgA and protective efficacy against influenza virus A [38]. IgA likely mediates protection through the neutralization of free virus particles [39] and/or intracellular killing and activation of an antiviral state of infected cells [40]. Whether the IgA response induced by RBD-TMC NPs follows these protective mechanisms is worth further investigation.

In addition to eliciting mucosal immune responses, nanoparticle-based vaccines also stimulate systemic immune responses. For example, oral vaccination with Omp-31 incorporated into TMC NPs significantly increased systemic humoral immune responses (e.g., IgG, IgG1 and IgG2a) [41]. Other studies also demonstrated that the intranasal delivery of TMC-loaded influenza antigens (4M2e and H3N2 influenza antigens) to mice strongly activated serum antibody responses [17,42]. Consistent with previous studies, we showed here that RBD-TMC NPs potently stimulated a systemic humoral immune response, as revealed by the upregulation of circulating IgG, IgG1, IgG2a and IgA, as well as neutralizing antibodies. This observation suggests that the systemic antibody response induced by our vaccine candidate through intranasal administration may not only prevent mucosal infection but also limit subsequent SARS-CoV-2 infections in the systemic compartment.

The strength and kinetics of the humoral immune response in SARS-CoV-2 patients have been extensively explored. It has been revealed that the breadth and magnitude of antibody responses correlate with disease severity, and patients with severe disease develop more antibodies, especially to the spike protein, compared with mild/moderate cases [43,44]. Furthermore, Seow et al. also demonstrated that neutralizing antibody titers peaked on Day 23 post-disease onset and rapidly decreased within 18–65 days [45]. A separate study revealed a rapid decline in the antibody response toward S protein within a 6-month period [46]. Consequently, the transient course of antibody response may create concern regarding the efficacy of vaccines that solely induce humoral immunity. In contrast, functional T cells robustly persisted and even increased over the time of observation [46]. Although the role of T cell responses in protecting against SARS-CoV-2 infection remains unclear, Peng et al. found that a greater proportion of SARS-CoV-2-specific CD8^+^ cells was detected in mild patients as compared with severe cases [47]. In this study, we monitored the intensity and diversity of T cells elicited in response to intranasal vaccination with RBD-TMC NPs. We found that this vaccine platform was capable of inducing both IFN-γ^+^CD4^+^ as well as IFN-γ^+^CD8^+^ cell responses. In particular, for CD8^+^ cells, the magnitude of CTL responses was stronger in RBD-TMC NP immunized mice, when compared with sRBD administration. This is in accordance with previous studies showing that RBD protein was immunogenic to splenic T cells in murine models in which the immunodominant epitopes of RBD corresponded to S_375-394_, S_405-469_, S_495-521_ and S_526-533_ [11,48]. The RBD used in our present study contained these T cell epitopes. The ability of TMC to augment the CTL response is not well understood. However, we postulate that TMC may promote endosomal escape into the cytosol, allowing the antigen to be further recognized and presented through an MHC-I-dependent pathway [49].

Regarding non-replicating vaccines, this vaccine formulation exhibited a safer profile than live vaccines. Thus, this platform of protein vaccine may be beneficial for a broad range of populations such as the elderly, children and immunocompromised individuals. Moreover, intranasal vaccination is a non-invasive procedure. Consequently, the vaccination could be performed with minimum medical resources or by less skilled personnel or even by self-immunization. This will facilitate mass immunization against SARS-CoV-2. However, the limitation of our vaccine platform is that multiple doses of immunization are required to obtain a breadth of immune responses. This can be improved by using an appropriate adjuvant.

## 5. Conclusions

**Figure 7:**
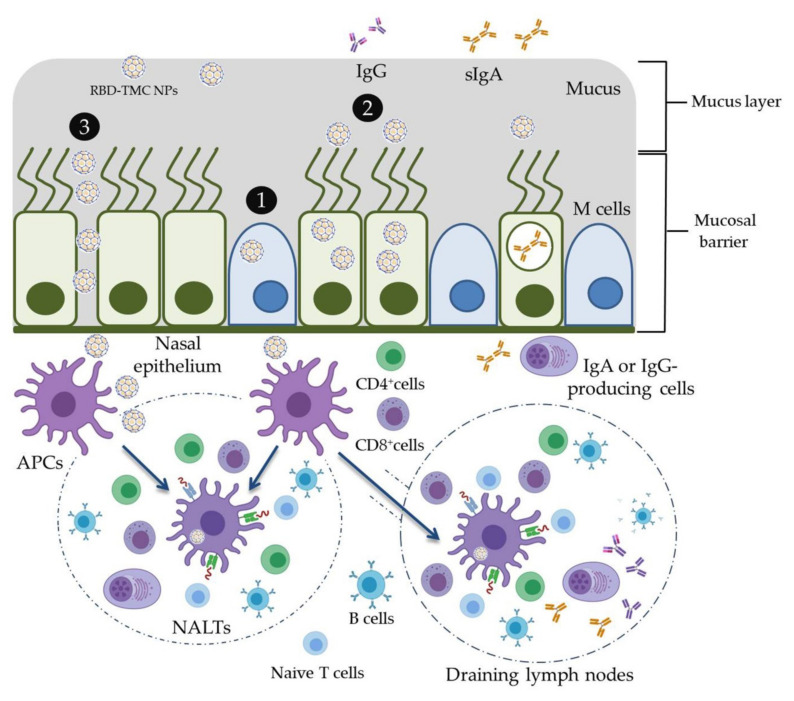
Schematic model of the potential working mechanisms of RBD-TMC NPs in the stimulation of immune responses. Upon entering into the respiratory tract, the cationic charges of TMC NPs facilitate the adhesion of NPs into the mucosa of the upper respiratory tract. Nanoparticles may then promote delivery of RBD into nasal cell populations by enhancing the uptake of NPs by (**1**) microfold cells (M cells) or (**2**) nasal epithelial cells, or (**3**) by stimulating transient opening epithelial cell junctions in which RBD-TMC NPs will be directly exposed to the immune cells underneath the mucosal barrier. Mucosal epithelial cells may process and present antigens to the immature DCs, while the particles taken up by M cells will be transcytosed and exposed to immune cells underneath the mucosal layer. Upon stimulation, immature DCs differentiate and migrate to nasal-associated lymphoid tissues (NALTs), where they present the antigens to both naïve and memory T cells, resulting in activation of the mucosal immune responses. In addition, DCs can migrate to draining lymph nodes, where stimulation of the systemic immune responses occurs.

In the present study, the mucoadhesive property of TMC was applied for the development of an intranasal SARS-CoV-2 vaccine. RBD was used as an immunogen and was encapsidated within TMC NPs. This vaccine platform was able to stimulate not only mucosal immunity but also systemic humoral and cell-mediated immune responses in mice upon intranasal immunization. The potential working mechanisms of RBD-TMC NPs in the induction of immune responses are proposed in Figure 7. These promising results drive us to develop RBD-TMC NPs or spike-TMC NPs into a nasal spray for human vaccination.

## Figures and Tables

**Table 1:** Physical properties of RBD-TMC NPs.

Nanoparticles	Particle Size (nm)	Polydispersity Index (PDI)	Zeta Potential (mV)	% Loading Efficiency (LE)
TMC NPs	380.3 ± 15.11	0.410 ± 0.028	16.0 ± 0.208	-
RBD-TMC NPs	386.5 ± 58.96	0.407 ± 0.019	12.9 ± 0.651	99.32 ± 1.18

## Data Availability

The data presented in this study are contained within the article.
